# Supplementary material for: Vitamin D Modified DSS-Induced Colitis in Mice via STING Signaling Pathway
Source: Biology (Basel). 2025 Jun 18;14(6):715. doi: 10.3390/biology14060715 (PMC12190092; doi:10.3390/biology14060715)
Supplement: Supplementary file 1 [file biology-14-00715-s001.zip › Supplementary Materials S1: 16s-report of gut microbiota/Groups/diff_analysis/TukeyHSD/Tukey_GBM_rotate.pdf]

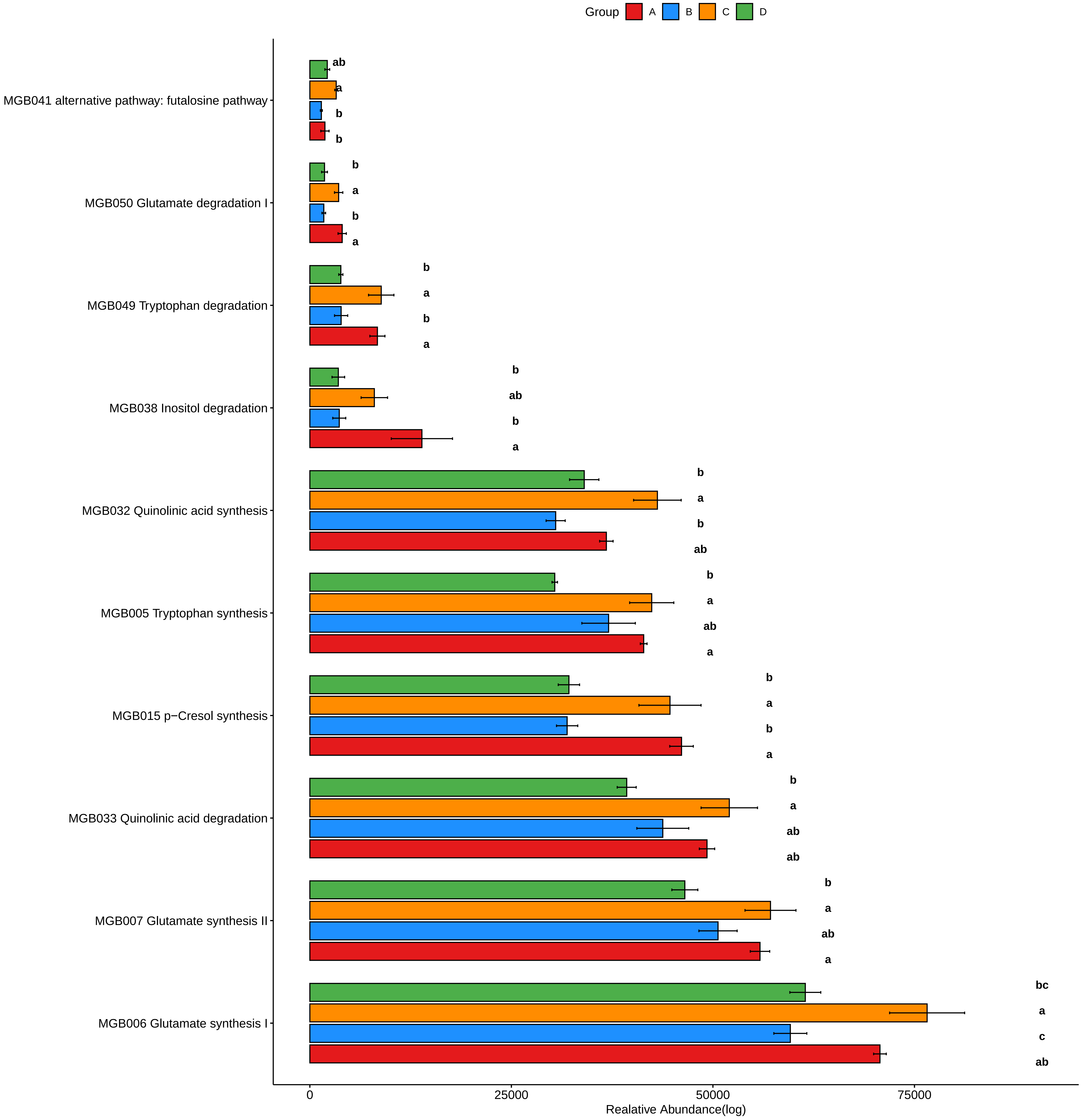

Group A B C D
MGB041 alternative pathway: futalosine pathway
ab
a
b
b
MGB050 Glutamate degradation I
b
a
b
a
MGB049 Tryptophan degradation
b
a
b
a
MGB038 Inositol degradation
b
ab
b
a
MGB032 Quinolinic acid synthesis
b
a
b
ab
MGB005 Tryptophan synthesis
b
a
ab
a
MGB015 p−Cresol synthesis
b
a
b
a
MGB033 Quinolinic acid degradation
b
a
ab
ab
MGB007 Glutamate synthesis II
b
a
ab
a
MGB006 Glutamate synthesis I
bc
a
c
ab
0
25000
50000
75000
Realative Abundance(log)
